# A case of autoimmune/inflammatory syndrome induced by adjuvants associated with breast augmentation: Coincidence or causality?

**DOI:** 10.1016/j.jdcr.2025.06.045

**Published:** 2025-07-21

**Authors:** Josefa Catalán Lobo, Vicente Orellana Westermeyer, Javier Arellano

**Affiliations:** a Department of Dermatology, Faculty of Medicine, University of Chile, Santiago, Chile; b Department of Dermatology, San Borja Arriaran Hospital, Santiago, Chile

**Keywords:** adjuvants, ASIA syndrome, autoimmune/inflammatory syndrome induced by adjuvants, autoimmunity, silicone

## Introduction

Autoimmune/inflammatory syndrome induced by adjuvants (ASIA syndrome) was first described in 2011 to unify under a single term the clinical manifestations observed in patients exposed to adjuvant substances such as silicone, aluminum hydroxide, squalene, silica, and infectious agents, among others.^1^^,^^2^ This syndrome can affect genetically predisposed individuals—most commonly women—following exposure to an exogenous substance. Clinically, it presents with heterogeneous and nonspecific symptoms, making diagnosis and treatment challenging.^3^

Most patients undergo multiple immunosuppressive therapies with inconsistent responses, with removal of the triggering adjuvant considered the definitive treatment.^3^^,^^4^

In this report, we describe the case of a patient with ASIA syndrome induced by silicone breast implants, who showed a favorable clinical response after explantation.

## Case report

**Fig 1:**
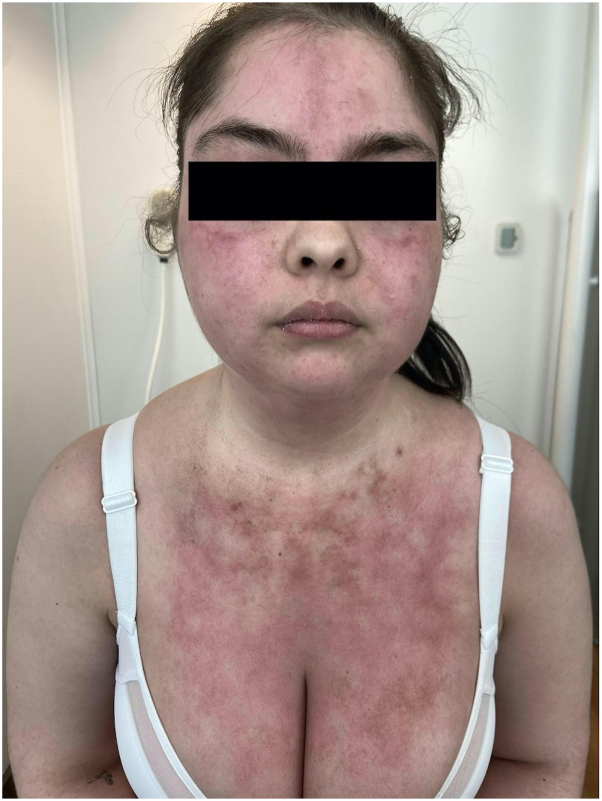
During medical treatment. Persistent erythema on the face and upper anterior chest.

A 25-year-old female with a family history of severe systemic lupus erythematosus has a sister who died due to complications of the disease. Her medical history included breast augmentation with silicone implants. Three years postsurgery, she began to experience constitutional symptoms, fatigue, intense facial erythema sparing the nasolabial folds, and a burning reticulated erythema over the upper chest (Fig 1).

Given the suspicion of systemic lupus erythematosus with cutaneous involvement versus amyopathic dermatomyositis, an autoimmune workup was conducted. Laboratory tests revealed positive antinuclear antibodies at a titer of 1:160, moderate anti-topoisomerase I antibody positivity, strong anti-Speckled Protein 100 antibody, moderate antinucleosome remodeling deacetylase complex subunit Mi-2 antibody, weak anti-centromere protein A antibody, and borderline levels of anti-threonyl-transfer RNA synthetase antibody and Signal Recognition Particle antibodies.

A skin biopsy showed superficial perivascular dermatitis with increased dermal mucin and melanosis, findings suggestive of connective tissue disease, leading to a diagnosis of undifferentiated connective tissue disease (UCTD).

She received topical corticosteroids and systemic oral prednisone up to 0.5 mg/kg/day. Azathioprine was initiated and escalated to 150 mg/day but discontinued after 1 month due to symptom exacerbation. Azathioprine was then replaced with mycophenolate mofetil 1.5 g/day for 4 months, which was also discontinued due to lack of clinical response.

Treatment was subsequently switched to hydroxychloroquine 200 mg/day, followed by the addition of cyclosporine at 1.3 mg/kg/day, which resulted in partial improvement of skin signs. However, systemic symptoms persisted, prompting the initiation of belimumab for 7 months, without significant response.

Due to continued disease activity, hydroxychloroquine was increased to 400 mg/day, and cyclosporine was replaced with methotrexate in an effort to manage the extensive cutaneous involvement.

Despite these sequential treatments, the patient continued to exhibit constitutional and cutaneous symptoms. It was then considered that the UCTD could be part of ASIA syndrome, induced by silicone.

**Fig 2:**
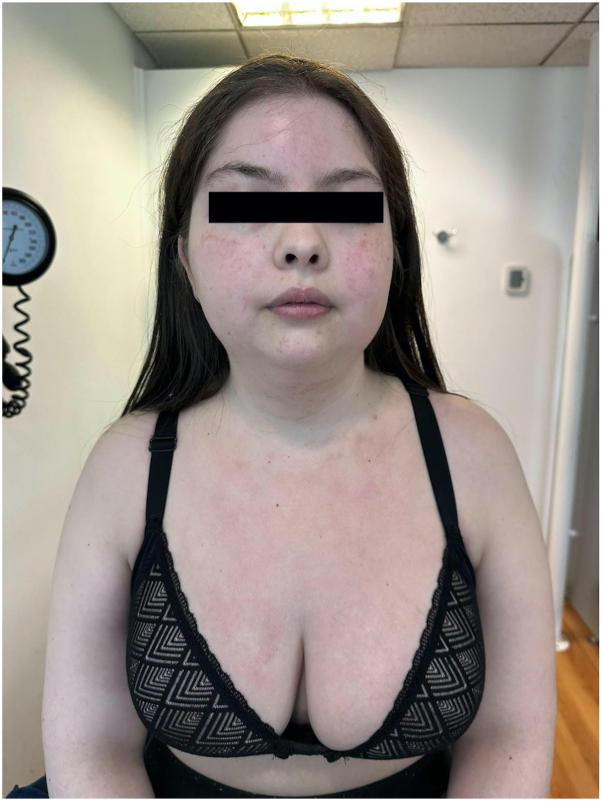
Follow-up after removal of silicone breast implants. Patient with minimal residual erythema on the face and upper anterior chest.

As a final therapeutic option, bilateral breast implant explantation was performed. The patient showed remarkable clinical improvement, with complete resolution of symptoms and significant reduction of erythema in sun-exposed areas by the fifth postoperative month. Laboratory values normalized, and all immunosuppressive medications were discontinued without relapse (Fig 2).

## Discussion

Although generally considered safe and inert, silicone breast implants have been implicated as potential inducers of autoimmune manifestations through mechanisms not yet fully elucidated. These likely involve immune hyperstimulation in genetically predisposed individuals.^5^^,^^6^

Silicone implants may trigger a specific immune response, leading to the release of proinflammatory cytokines that activate T and B lymphocytes. Moreover, they may enhance immunoreactivity by interacting with connective tissue components such as mucopolysaccharides.^3^^,^^7^

Several risk factors have been proposed to increase susceptibility to ASIA syndrome, including a history of allergic or autoimmune disease, smoking, obesity, and vitamin D deficiency.^8^

The clinical onset of ASIA syndrome typically occurs between 1 month and 5 years after exposure to an adjuvant and may be difficult to diagnose due to nonspecific symptoms. Common manifestations include arthralgia (73.8%), chronic fatigue (65.6%), myalgia (50.8%), sleep disturbances (43.8%), weakness (49.4%), and fever (38.8%). Up to 54.4% of patients test positive for autoantibodies, most commonly antinuclear antibodies (48.2%).^6^^,^^9^

Among the diseases most frequently associated with ASIA syndrome are UCTD (38.8%) and Sjögren’s syndrome (16.8%).^3^^,^^9^

Due to the nonspecific nature of the syndrome, diagnosis requires fulfillment of at least 2 major criteria or 1 major and 2 minor criteria (Table I).^1^^,^^2^

**Table I:** Major and minor diagnostic criteria for ASIA syndrome. Diagnosis is established by the presence of at least 2 major criteria or 1 major and 2 minor criteria

Major criteria
Prior exposure to an adjuvant
Chronic fatigue and/or sleep disturbances
Myalgia and/or muscle weakness
Arthritis and/or arthralgia
Cognitive impairment and/or memory loss
Peripheral neurological manifestations
Dry mouth and/or dry eyes
Neurological manifestations
Pyrexia
Improvement after adjuvant removal
Typical biopsy of affected organs
Minor criteria
Antibodies against the suspected adjuvant
HLA-DRB1 and/or HLA-DQB1 positivity
Evolution into an autoimmune disease
Other clinical manifestations (eg, irritable bowel syndrome, Raynaud’s phenomenon)

Adapted from Shoenfeld and Agmon-Levin (2011).^1^

*ASIA*, Autoimmune/inflammatory syndrome induced by adjuvants.

In cases related to silicone implants, symptom improvement or resolution has been reported in 50% to 98% of patients following explantation. Several factors may influence outcomes, including implant characteristics, duration of implantation, disease features, comorbidities, type of explant surgery, and whether postexplant reconstruction was performed.^2^^,^^6^^,^^9^

In this case, the patient fulfilled 4 major criteria: chronic fatigue, temporal association between symptom onset and adjuvant exposure, neuropathic burning pain, and symptom resolution only after adjuvant removal.^10^

In conclusion, when clinical suspicion of ASIA syndrome is high, the removal of the causative adjuvant should be considered when feasible.

## Conflicts of interest

None disclosed.
